# *Persicimonas caeni* gen. nov., sp. nov., the Representative of a Novel Wide-Ranging Predatory Taxon in *Bradymonadales*

**DOI:** 10.3389/fmicb.2020.00698

**Published:** 2020-04-22

**Authors:** Shuo Wang, Dashuai Mu, Zong-Jun Du

**Affiliations:** ^1^Marine College, Shandong University, Weihai, China; ^2^State Key Laboratory of Microbial Technology, Shandong University, Qingdao, China

**Keywords:** *Bradymonadales*, *Bradymonadaceae*, 16S rRNA gene, phylogenetic analysis, predatory bacteria, genomic analysis

## Abstract

A novel bacterial strain, designated YN101^T^, was isolated from a marine solar saltern in the coast of Weihai, Shandong Province, China. Strain YN101^T^ was Gram-stain negative, facultatively anaerobic, oxidase and catalase negative bacterium with the ability to prey on other microbes. A cross-streaking culture method was utilized to analyze the predatory activity of strain YN101^T^. The results showed strain YN101^T^ could prey on various bacteria, either Gram-stain negative or Gram-stain positive. According to the predatory assays, different species in the same genus may behave differently when attacked by strain YN101^T^. The predatory behavior of strain YN101^T^ to four typical species was analyzed, and furthermore, predation to *Algoriphagus marinus* am2^T^ were quantitatively studied by fluorogenic quantitative PCR, and the gene copies decreased over two magnitudes. Phylogenetic analysis based on 16S rRNA gene sequences indicated that strain YN101^T^ shared the greatest sequence similarity of 93.9% to *Bradymonas sediminis* FA350^T^. The complete genome sequence of strain YN101^T^ was 8,047,306 bp in size and the genomic DNA G + C content was 63.8 mol%. The digital DNA-DNA hybridization (dDDH) values and average nucleotide identity (ANI) values between strain YN101^T^ and *B. sediminis* FA350^T^ were 13.9 and 74.0%. The genetic features showed that the biosynthesis of many important compounds was deficient in genome of strain YN101^T^, which may lead to its predation. Moreover, its genome encoded many genes affiliated with type IV pili, secretion system, membrane proteins and transduction proteins. Similar with myxobacteria and *Bdellovibrio* and like organisms (BALOs), these genes should play important roles in motility, adhesion or virulence to attack prey cells during predation. The predominant polar lipid profile of strain YN101^T^ consisted of phosphatidylethanolamine (PE), phosphatidylglycerol (PG), diphosphatidylglycerol (DPG), and one unidentified aminophospholipid (APL). The major cellular fatty acid of strain YN101^T^ was iso-C_17:0_, and the sole respiratory quinone was MK-7. Based on the chemotaxonomic, physiological and biochemical characteristics, strain YN101^T^ represents a novel species of a novel genus in the family *Bradymonadaceae*, for which the name *Persicimonas caeni* gen. nov., sp. nov. is proposed. The type strain is YN101^T^ (=KCTC 72083^T^ = MCCC 1H00374^T^).

## Introduction

As of 2019, there are three valid published species separated into two genera in the order *Bradymonadales*, including *Bradymonas sediminis* FA350^T^ (Wang et al., 2015), *Lujinxingia litoralis* B210^T^ and *Lujinxingia sediminis* SEH01^T^ (Guo et al., 2019). *B. sediminis* FA350^T^ is the representative of the type genus of the type family. However, with developments in metagenome and 16S rRNA gene high-throughput sequencing technologies, more previously uncultured members of *Bradymonadales* have been discovered (Cheng et al., 2018; Bachran et al., 2019).

Members of the order *Bradymonadales* have numerous common properties, including low growth rate and lawn rather than colony formation on plates, etc. Furthermore, they all prey on other bacteria. As a new predatory order in *Deltaproteobacteria*, *Bradymonadales* were considered facultative predators according to our previous studies (Wang et al., 2019). Bacterial predators are artificially divided in two groups: facultative and obligate predators (Pérez et al., 2016). Facultative predators are mainly represented by myxobacteria, which can prey on bacteria or utilize other nutrients and substances from the environment. *Bdellovibrio bacteriovorus* is one representative of the obligate predators, which belongs to class *Oligoflexia* (Dashiff et al., 2011; Soo et al., 2015). For both forms of predation, hunting strategies can be categorized into two general types: epibiotic predation and direct invasion or penetration of the periplasm (Pérez et al., 2016). During the attack phase of the epibiotic predator, genes implicated in chemotaxis and flagellum biosynthesis were highly expressed, whereas during the attachment phase, genes involved in protein secretion and housekeeping genes were dramatically upregulated (Pérez et al., 2016). *Bdellovibrio exovorus* are highly motile cells during the extracellular attack phase, which swim using flagella to encounter their prey (Sockett, 2009; Rotem et al., 2015). During the growth phase, the predator irreversibly attaches to a Gram-stain negative bacterium and penetrates the periplasmic space, where it grows, replicates and kills the prey cell (Pérez et al., 2016). Almost all previously reported bacterial predation occurs in groups, including the two aforementioned types. However, the lysed cells after group attack benefit both the predator secreting cells and the non-secreting cells. Cells of *Myxococcus xanthus*, a social bacterium predator, move in search of prey in a coordinated manner, forming dynamic multicellular groups referred to as swarms (Muñoz-Dorado et al., 2016), and type IV pili are associated with this strategy (Yoshikawa et al., 2008). Bacterial predation is thus related to different systems of motility.

Studies on *B. sediminis* FA350^T^ have described its predation strategy of cooperative attack. Genomic analyses showed cells encoded with genes affiliated with type IV pili, which allow cells to swiftly glide to prey during predation (Wang et al., 2019). *B. sediminis* FA350^T^ are able to prey on a wide range of different types of bacteria, with some presently unknown selection rules. Results of phenotypic tests and plate counting demonstrated the predation of *B. sediminis* FA350^T^, however, quantification of the predation over different time periods with mixed cultures remained elusive. Here, we introduce type strain YN101^T^, a novel predatory taxon of *Bradymonadales*. Predation differences between strain YN101^T^ and *B. sediminis* FA350^T^ were studied systematically. As the representatives of predatory groups in *Deltaproteobacteria*, *Myxococcus xanthus* DK1622^T^ was chosen as related strain. Two genomes of *Bdellovibrio* were also analyzed compared with strain YN101^T^. Study on this novel species will further expand the membership of *Bradymonadales*, a new predatory bacterial group, which is of great significance in the exploration of microbial resources. *Bradymonadales* distributes widely in marine environment and may play important roles in the composition of marine microbial community and the global biochemical cycle. Descriptions on metabolic features during predation according to comparative genomic analyses are also important for deep insights into predatory mechanism. Therefore, studies on strain YN101^T^ will be of great significance in many aspects.

## Materials and Methods

### Bacteria Strains

Strain YN101^T^ was isolated from a sediment sample collected from a marine solar saltern (122°14′E, 36°54′N) in Weihai, China. A 100 μL aliquot of diluted sample was spread on marine agar 2216 (MA; BD) using the standard dilution plating technique to obtain pure cultures. Plates were incubated at 30°C for more than 30 days, and strain YN101^T^ was isolated and stored at −80°C in sterile 15 % (v/v) glycerol supplemented with 1% (v/v) NaCl. *B. sediminis* FA350^T^ isolated by our laboratory (Wang et al., 2015) was used as the related strain for phenotypic and genetic analyses.

### 16S rRNA Gene Sequence Analyses

The 16S rRNA gene of strain YN101^T^ was amplified from the genomic DNA by PCR with the universal primers 27F and 1492R (Liu et al., 2014). PCR products were purified using a PCR product purification kit (Takara Bio Inc., Kusatsu, Japan), then ligated to the pMD18-T vector (Takara Bio Inc.) according to the manufacturer’s instructions. Sequencing was performed by Ruibiotech Co. Ltd (Qingdao, China) using universal primers M13-R and M13-F (Han et al., 2017a). Phylogenetic trees were built by neighbor-joining, maximum-likelihood, and maximum-parsimony methods using MEGA 7.0 software according to the 16S rRNA gene sequences (Felsenstein, 1981; Saitou and Nei, 1987; Kannan and Wheeler, 2012; Kumar et al., 2016). Genetic distances were calculated using the Kimura two-parameter model (Kimura, 1980). The BLAST algorithm was used to search for similar sequences^1^. The EzTaxon-e server^2^ was used to acquire sequence similarity values (Kim et al., 2012).

### Morphological Observations and Physiological Tests

The effects of different growth temperatures were tested on incubations at 4, 8, 15, 20, 25, 28, 30, 33, 37, 40, 45, 50, and 55°C on modified MA until growth was indicated by visible lawn (no obvious colonies formed, about end-logarithmic growth period). Growth conditions were recorded every 12 h. Modified MA consisted of distilled water with the following additions (all g L^–1^): sea salt (Sigma-Aldrich, St. Louis, MO, United Stated), 30; yeast extract, 1; peptone, 5; sodium pyruvate, 0.1; and agar, 18. The pH was adjusted to 7.5. The effects on growth induced by pH were tested by altering the pH of modified MB without agar using various buffers: MES (pH 5.5 and 6.0), PIPES (pH 6.5 and 7.0), HEPES (pH 7.5 and 8.0), Tricine (pH 8.5), and CAPSO (pH 9.0 and 9.5) at concentrations of 20 mM (Sangon Biotech Co., Ltd., Shanghai, China). The pH of the medium was adjusted by adding 1M HCl or NaOH before autoclaving and OD_600_ values of the culture were measured after incubation. The growth under different NaCl concentrations from 0 to 12% NaCl at 1% intervals was assessed using modified MA without sea salt, in which purified water was substituted for artificial seawater (0.32% MgSO_4_, 0.12% CaCl_2_, 0.07% KCl, and 0.02% NaHCO_3_, all in w/v).

The morphological and physiological features of strain YN101^T^ in end-logarithmic growth period were examined after incubation at 37°C. Motility was examined using the hanging-drop method and gliding on modified MB supplemented with 0.3% agar (Bernardet et al., 2002). A transmission electron microscope (JEM-1200EX; JEOL Ltd., Tokyo, Japan) was used to observe cell size and morphology. Antibiotic sensitivity was assessed as described by the Clinical and Laboratory Standards Institute (CLSI, 2012): a cell suspension (McFarland standard 0.5) was swabbed over the surface of modified MA plates to create a uniform lawn before aseptic placement of antibiotic disks onto the agar surface.

Catalase activity was detected by bubble production after the addition of 3% (v/v) H_2_O_2_, and oxidase activity was determined using reagent kit (Diamond Bio Scientific LLC). Anaerobic growth was determined by inoculation on modified MA supplemented with 0.1% (w/v) sodium pyruvate, with or without 0.5% (w/v) sulfate or 0.1% (w/v) nitrate in an anaerobic jar. Modified MB supplemented with 0.1% (w/v) nitrate was used for the nitrate-reducing test; inoculated and uninoculated cultures in test tubes under aerobic and anaerobic conditions at 37°C served as treatment and control groups, respectively. Hydrolysis of starch, cellulose, lipids and alginate was tested on modified MA supplemented with 0.2% (w/v) soluble starch, 0.5% (w/v) CM-cellulose, 1% (w/v) Tweens 20, 40, 60, 80, and 2% (w/v) sodium alginate (Cowan and Steel, 1965).

### Chemotaxonomic Characteristics

Respiratory quinones were analyzed by log-phase cells under optimal physiological conditions. Extraction from 300 mg of freeze-dried cell material was separated into different classes by TLC on silica gel (Tindall B., 1990; Tindall B.J., 1990). In accordance with the spots on the silica gel plate, the effective components were removed from the plate and analyzed further by using reverse-phase HPLC (VP-ODS, 250 × 4.6 mm; Shimadzu, LC-20A), then eluted with a mixture of acetonitrile and isopropanol (3:2, v/v) at a flow rate of 1 mL min^–1^.

Fatty acid composition was determined by first incubating cells of strain YN101^T^ on modified MB at 37°C to reach the exponential stage of growth, according to the four quadrants streak method. Fatty acids were saponified, methylated and extracted using the standard protocol of the Sherlock microbial identification system version 6.1 (MIDI Inc., Newark, United States) equipped with an Agilent model 6890N gas chromatograph (Agilent Technologies, Santa Clara, CA, United States). Peaks were automatically integrated and fatty acid names and percentages were calculated using MIS standard software with the TSBA40 database (MIDI Inc.).

Cells cultured in modified MB at 37°C were used to determine the polar lipids using two-dimensional silica gel thin-layer chromatography (TLC, 1.05554.0001; Merck, Kenilworth, United States) (Xu et al., 2007). Total lipid materials were detected using molybdatophosphoric acid and specific functional groups were detected using spray staining reagents (Sigma-Aldrich) on four separate TLC plates: phosphomolybdic acid solution (total lipids), molybdenum blue solution (phosphates), *α*-naphthol sulfuric solution (carbohydrates) and ninhydrin (amines).

Tests for other physiological and biochemical characteristics were determined using API 20E and API ZYM test strips (bioMérieux Inc., Marcy-l’Étoile, France), according to the manufacturer’s instructions, apart from the suspension being prepared with sterilized 3% (w/v) sea salt (Sigma-Aldrich) solution. The strain was further tested for oxidization ability of various carbon sources using the GEN III Microplate according to the manufacturer’s instructions (Biolog Inc., Hayward, CA, United States). The ability to produce acid from carbohydrates was tested using the API 50CHB fermentation kit (bioMérieux Inc.) according to the manufacturer’s instructions. The salinity of the supplementary medium of both kits was adjusted to 3% with sea salt (Sigma-Aldrich). The API strips and Biolog results were recorded every 24 h after incubation at 37°C until all reactions were steady. All API and Biolog tests were performed in duplicate under identical conditions.

### Predation Tests

Cross-streaking incubation tests (Supplementary Figure S1B) were performed to demonstrate the predation of strain YN101^T^ and *B. sediminis* FA350^T^. A suspension (McFarland standard 5.0) of predatory bacteria was prepared and inoculated on MA in the first direction, followed by the suspension of prey bacteria (McFarland standard 5.0) inoculated in the second direction across the first inoculation line. The plate was then incubated until the growth of both bacteria was indicated by visible lawn.

To further detect the predation of *Bradymonadales*, a co-cultured system allowing predation was built. Concentrated suspensions (McFarland standard 5.0) of strain YN101^T^ (predator) and *Algoriphagus marinus* am2^T^ (prey) in logarithmic phase were co-incubated (1:1 volume ratio) onto the micropore filtration membrane (50 mm, 0.2 μm) attached to the MA medium at 33°C (Han et al., 2017b). Mono-cultured predator and prey were incubated as control groups. Genomic DNA was extracted from the inoculated cultures after 0, 68, and 120 h. Primers and TaqMan probes (modified with FAM/BHQ1) based on specific gene sequences for each species were designed using the PrimerQuest Tool^3^. Changes in biomass of each species in the co-cultured system were quantified by fluorescent quantitative realtime-PCR. Results were demonstrated as line graphs using Origin 8.0 Trial software^4^. The PCR and reaction system program, as well as the sequences of specific genes, corresponding primers and TaqMan probes are available in Supplementary File S1 in the online Supplementary Material.

### Whole Genome Sequencing and Analyses

The genomic DNA of strain YN101^T^ was extracted using a DNA extraction kit (Takara Bio) according to the manufacturer’s instructions. The complete genome of strain YN101^T^ was sequenced by Novogene Biotechnology Co. (Beijing, China) using the Illumina PE150 platform (Illumina, San Diego, CA, United States). Assembly of the raw sequencing data was performed using SOAPdenovo version 2.04^5^, SPAdes and ABySS, and was finally integrated by CISA. The assembly results were optimized with GapCloser Version 1.12, and fragments below 500 bp were filtered out. A phylogenomic tree was constructed based on 92 up-to-date bacterial core gene sequences of strain YN101^T^ and type strains of related taxa using the UBCG tools^6^ (Na et al., 2018) with a maximum-likelihood algorithm (Felsenstein, 1981). The digital DNA-DNA hybridization (dDDH) values between strain YN101^T^ and the related strains were calculated using the Genome-to-Genome distance calculator (GGDC)^7^. Evaluations and statistical analyses were undertaken to perform genetic predictions. Comparative genetic analyses were performed utilizing RAST (Rapid Annotation using Subsystem Technology^8^) and KEGG (Kyoto Encyclopedia of Genes and Genomes)^9^ software. The analyses were presented using Heml 1.0 heatmap illustration software^10^.

## Results

### 16S rRNA Gene Sequence Analyses

**FIGURE 1:**
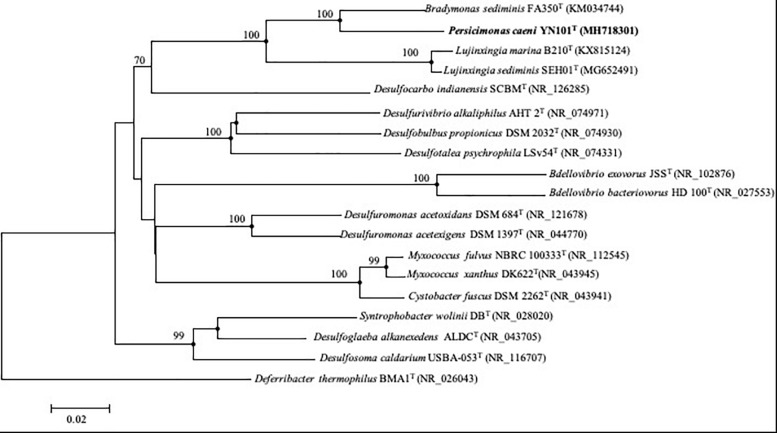
Phylogenetic tree constructed from 16S rRNA gene sequences, showing the position of strain YN101^T^ and type strains of related taxa within the phylum *Proteobacteria*. The tree was constructed using the neighbor-joining algorithm. The filled circles indicate that the corresponding nodes were also found in the trees generated with the maximum-likelihood and maximum-parsimony algorithms. Bootstrap values (expressed as percentages of 1000 replications) > 70% are shown at branch points. GenBank accession numbers of 16S rRNA gene sequences are given in parentheses. *Deferribacter thermophilus* BMA1^T^ (NR_026043) was used as an outgroup. Bar, 0.02 substitutions per nucleotide position.

A nearly complete 16S rRNA gene sequence of strain YN101^T^ obtained by amplification (1458 bp) was included in the 16S rRNA gene sequence assembled from genomic sequences (1536 bp). All three 16S rDNA sequences from the genome sequence were of the same lengths and could be identified as complete sequences in accordance with the NCBI gene database^11^. Strain YN101^T^ shared the highest level of similarity with *B. sediminis* FA350^T^ (93.9%), followed by *Pelobacter propionicus* DSM 2379^T^ (85.0%), *Desulfuromonas svalbardensis* 112^T^ (85.0%) and *Geobacter pickeringii* G13^T^ (85.0%), all of which belong to the order *Desulfuromonadales*. On the basis of phylogenetic analysis using the neighbor-joining algorithm, strain YN101^T^ formed a phylogenetic cluster with *B. sediminis* FA350^T^ at the 100% bootstrap confidence level, forming an evident cluster group distinctly separated from genus *Lujinxingia*. The taxonomic status was also demonstrated by maximum-likelihood and maximum-parsimony trees (Figure 1).

### Morphological Observations and Physiological Tests

Optimal growth of strain YN101^T^ occurred with 3–4% NaCl (range 1–10%, w/v), at 37°C (range 15–50°C) and pH 8.0–8.5 (range 6.5–9.0). However, *B. sediminis* FA350^T^ grow at 33°C (20–37°C) optimally. Few apparent colonies formed after 6 days (logarithmic phase) of incubation at 37°C, and the colonies were circular or irregular, pink-colored, about 1 mm diameter, and with entire and transparent edges. Electron micrograph images showed rod-shaped cells for strain YN101^T^ (0.3–1.0 × 1.5–5.0 μm) with unambiguous flagella (Supplementary Figure S2A). Rod-shaped cells with pili are for *B. sediminis* FA350^T^ (Wang et al., 2019). Motile cells of strain YN101^T^ used flagella and gliding, which differed from cells of *B. sediminis* FA350^T^ that can only weakly glide (Supplementary Figure S2B). Cells of strain YN101^T^ were mobile over a wider range and penetrated their prey more deeply (*Pseudorhodobacter ferrugineus* JCM 20687^T^; Uchino et al., 2002) than *B. sediminis* FA350^T^. *M. xanthus* DK1622^T^ are motile by means of pili (Sun et al., 2000), whereas members of *Bdellovibrio* move using flagella (Lambert et al., 2006).

Inoculated plates were incubated at 37°C for up to 6 days. Cells of strain YN101^T^ were shown to be resistant to vancomycin (30 μg), kanamycin (30 μg) and tobramycin (10 μg), and were susceptive to lincomycin (2 μg), norfloxacin (30 μg), penicillin (10 μg), neomycin (30 μg), clindamycin (30 μg), tetracycline (30 μg), ceftriaxone (30 μg) and erythromycin (15 μg). The related strain, *B. sediminis* FA350^T^, was shown to be extremely sensitive to penicillin (10 μg), erythromycin (15 μg), chloromycetin (30 μg), clarithromycin (15 μg), rifampicin (5 μg) and cefotaxime (30 μg), and were resistant to tetracycline (30 μg), trimethoprim (5 μg), nalidixic acid (30 μg) and vancomycin (30 μg).

### Chemotaxonomic Characteristics

Strain YN101^T^ was shown to utilize nitrate but not sulfate as an electron acceptor for anaerobic respiration, which was consistent with *B. sediminis* FA350^T^. Both strain YN101^T^ and *B. sediminis* FA350^T^ tested negative for oxidase activity, but positive for catalase and nitrate reduction. Iso-C_17:0_ (54.8%) was the only major fatty acid detected in strain YN101^T^, and other component of fatty acids were lower than 10.0%. However, *B. sediminis* FA350^T^ contained three major fatty acids, including iso-C_15:0_ (40.9%), C_17:0_ (30.7%) and iso-C_17:1_ *ω*10*c* (13.7%). Further details differentiating strain YN101^T^ from *B. sediminis* FA350^T^ are shown in Table 1 and Supplementary Table S1. The sole respiratory quinone of strain YN101^T^ was MK-7, which concurred with the related strain.

**TABLE 1:** Phenotypic characteristics that distinguish *Persicimonas* from the neighbor genera.

Characteristics	1	2	3^§^
Habitat	Saltern sludge	Marine sediment	Marine sediment
Colony color	Pink	Orange-red	Orange
Cell size (μm)	0.5 × 1.0–2.5	0.3–1.0 × 1.5–5.0	0.3–0.5 × 1.8–5.1
Means of motility	Flagella and gliding	Weakly gliding	Gliding
Temperature Range (°C)	15–50	20–37	20–42
NaCl tolerance (%, w/v)	1–10	1–9	0–8
Catalase reaction	–	+	+
Nitrate reduction	–	+	–
Arginine hydrolase	+	–	–
D-glucose oxidation	+	–	+
Tryptophan deaminase	–	+	+
Major fatty acids	Iso-C_17:0_	Iso-C_17:1_*ω*10*c*, C_15:0_, iso-C_17:0_	Iso-C_15:0_ and iso-C_14:0_ 3-OH
Major polar lipids	DPG, PG, PE, APL	DPG, PG, PE	DPG, PG, PE, L
Genome size (Mbp)	8.0	5.0	5.0–5.3
G + C content (mol%)	63.8	61.1	63.6–64.7

*1, *Persicimonas*; 2, *Bradymonas*; 3, *Lujinxingia*. All genera were rod-shaped and Gram-stain negative bacteria, negative for oxidase, and contained MK-7 as sole respiratory quinone. **^§^** Data from Guo et al. (2019).*

The major polar lipids detected in strain YN101^T^ were phosphatidylglycerol (PG), phosphatidylethanolamine (PE), diphosphatidylglycerol (DPG), and an unidentified aminophospholipid (APL). Additionally, an unidentified phosphatidylcholine (PC) was detected in moderate or minor amounts (Supplementary Figure S3). PG, PE, and DPG were detected as the major polar lipids in *B. sediminis* FA350^T^, and unidentified APL as well as PC was detected in moderate and or minor amounts. Therefore, the polar lipid profiles differed between strain YN101^T^ and the genus *Bradymonas*. Further details of the polar lipid contents of strain YN101^T^ and the related strain *B. sediminis* FA350^T^ are shown in Supplementary Figure S3. Other details differentiating strain YN101^T^ and the related genera are listed in Table 2.

**TABLE 2:** Phenotypic characteristics that distinguish *Persicimonas caeni* YN101^T^ from *Bradymonas sediminis* FA350^T^.

Characteristics	1	2
**Enzyme activities**		
Esterase (C4)	–	+
Lipase (C14)	–	+
Valine arylamidase	–	+
Cystine arylamidase	–	+
Trypsin	–	+
Acid phosphatase	+	–
Arginine hydrolase	+	–
Tryptophan deaminase	–	+
**Carbon oxidation**		
D-Raffinose	–	+
*α*-D-Glucose	–	+
*α*-Keto-Glutaric acid	+	–
Acetic Acid	–	+
**Acids production**		
D-Fructose	+	–
L-Sorbose	+	–
D-Lyxose	+	–

*1, *Persicimonas caeni* YN101^T^; 2, *Bradymonas sediminis* FA350^T^. The type strains of both genera were positive for alkaline phosphatase, esterase lipase (C8), leucine arylamidase, naphthol-AS-BI-phosphohydrolase and gelatinase; produced acids from D-ribose, esculin, D-tagatose and potassium 5-ketogluconate; oxidized D-glucuronic acid, glucuronamide and acetoacetic acid. +, Positive; –, negative.*

### Phenotypic Characteristics of Predation

The predatory range of strain YN101^T^ was investigated for activity against 27 different species of bacteria isolated from marine and saltern environments, which included Gram-stain negative bacteria and Gram-stain positive bacteria (data not shown). Four representative species were selected for further analyzed, including three Gram-stain negative and one Gram-stain positive species. Especially, *Woeseiaceae* and *Nocardioides* were the representatives of sulfur-metabolism and antibiotic-production groups (Mußmann et al., 2017; Ortiz-López et al., 2018). These functional bacteria groups were the focus on studies about global material circulation and new sources exploration of antibiotics, recently. Especially, bacterial predations can influence the variations and abundances of microorganism (Pérez et al., 2016). Studies on these preys could help discuss about effects of predation on natural material circulations.

After cross-streaking incubation of strain YN101^T^ and the test bacteria, plates were incubated until the growth of both bacteria was indicated by visible lawn (Supplementary Figure S1A). When strain YN101^T^ preyed on test bacteria like *Nocardioides gilvus* XZ17^T^ (Zhang et al., 2016), *Woeseia oceani* XK5^T^ (Du et al., 2016) and *A. marinus* am2^T^, the predator could be detected by 16S rRNA gene sequencing in the second line (Supplementary Figure S1B), but the test bacteria not. However, when the predator did not feed on the test bacteria, such as *Algoriphagus resistens* NH1^T^ (Han et al., 2017c), strain YN101^T^ could only be detected in the first line. Results of cross-streaking tests incubating *B. sediminis* FA350^T^ and the same test bacteria are also shown in Supplementary Figure S1A. Predation occurred with cell-to-cell attachment, and biofilm formation could be observed during the whole process.

**FIGURE 2:**
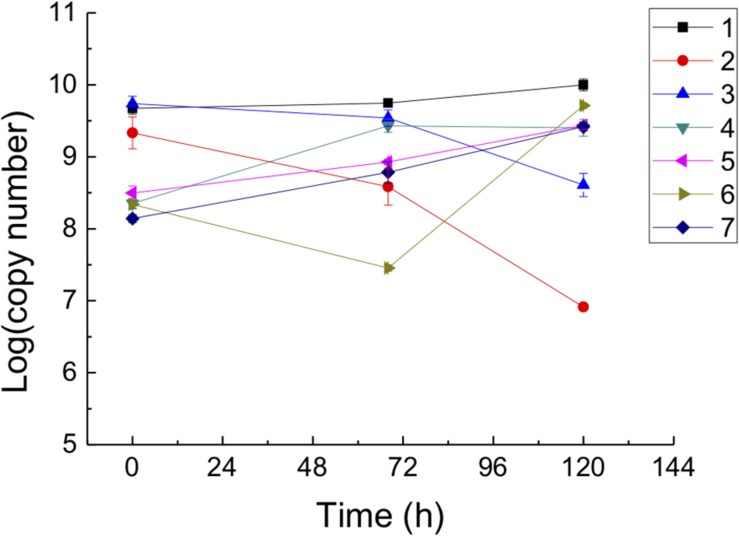
Biomass quantifying of strain YN101^T^, *B. sediminis* FA350^T^ and prey *Algoriphagus marinus* am2^T^ mono-cultured and co-cultured for different time (0, 68, and 120 h) using fluorogenic quantitative PCR. Horizontal axis represented incubation time, and vertical axis represented the logarithm of gene copy number. 1, *A. marinus* am2^T^ (RuBisCO); 2, *P. caeni* YN101^T^ and *A. marinus* am2^T^ (RuBisCO); 3, *B. sediminis* FA350^T^ and *A. marinus* am2^T^ (RuBisCO); 4, *P. caeni* YN101^T^ (FlgB); 5, *B. sediminis* FA350^T^ (YpfJ); 6, *P. caeni* YN101^T^ and *A. marinus* am2^T^ (FlgB); 7, *B. sediminis* FA350^T^ and *A. marinus* am2^T^ (YpfJ). Each design was repeated three times, and the result was the average of the three replicates.

Biomass fluctuations of predators, strain YN101^T^and *B. sediminis* FA350^T^, and prey, *A. marinus* am2^T^, after mono- and co-culture for 0, 68, and 120 h are demonstrated in Figure 2. The gene copy number of prey co-cultured with strain YN101^T^ decreased from 1.9 × 10^9^ to 7.9 × 10^6^ copies/μL at 120 h. However, cell numbers of the mono-cultured prey showed no significant change. Order of magnitudes of both mono- and co-cultured strain YN101^T^ increased by approximately 1.5, but growth of the co-cultured cells was slightly larger, indicating that the predator strain YN101^T^ grew by killing prey *A. marinus* am2^T^. The gene copy number of *A. marinus* am2^T^ decreased from 5.0 × 10^9^ to 3.9 × 10^8^ copies/μL at 120 h when co-cultured with *B. sediminis* FA350^T^. Co-cultured *B. sediminis* FA350^T^ had a consistent increase in logarithmic gene copy number values, with greater amplification than for mono-cultured cells. The above results implied that strain YN101^T^ and *B. sediminis* FA350^T^ are bacterial predators. Quantitative standard curves of specific genes are shown in Supplementary Figure S7, and data from fluorescent quantitative PCR are shown in Supplementary File S2.

The predatory features were summarized as below. Strain YN101^T^ predated a wide range of bacterial species, no matter Gram-stain negative or Gram-stain positive bacteria, which was similar with myxobacteria. Chemotactic predation by gliding occurred in strain YN101^T^ when living on surface, but not very extremely as myxobacteria. Predation depended on cell-to-cell contact, and predation in liquid was not observed. Details of predatory features were list in Table 3.

**TABLE 3:** Predatory features between strain YN101^T^ and each type species of related genera.

Predatory features	1	2	3	4^§^	5^§^
Facultative/obligate predators	F	F	F	O	O
Range of prey	G^+^ and G^–^	G^+^ and G^–^	G^+^ and G^–^	G^–^	G^–^
Chemotactic predation	W	W	+	+	+
Predating strategy	Epibiotic	Epibiotic	Group attack	Periplasmic	Epibiotic
Predation in liquid	–	–	+	+	+

*1, Strain YN101^T^; 2, *Bradymonas sediminis* FA350^T^; 3, *Myxococcus xanthus* DK1622^T^; 4, *Bdellovibrio bacteriovorus* HD100^T^; 5, *Bdellovibrio exovorus* JSS^T^. +, Positive; –, negative; G^+^, Gram-positive; G^–^, Gram-negative; W, weakly. **^§^** Data from Pérez et al. (2016).*

### Genome Data and Comparative Genomic Analysis

**FIGURE 3:**
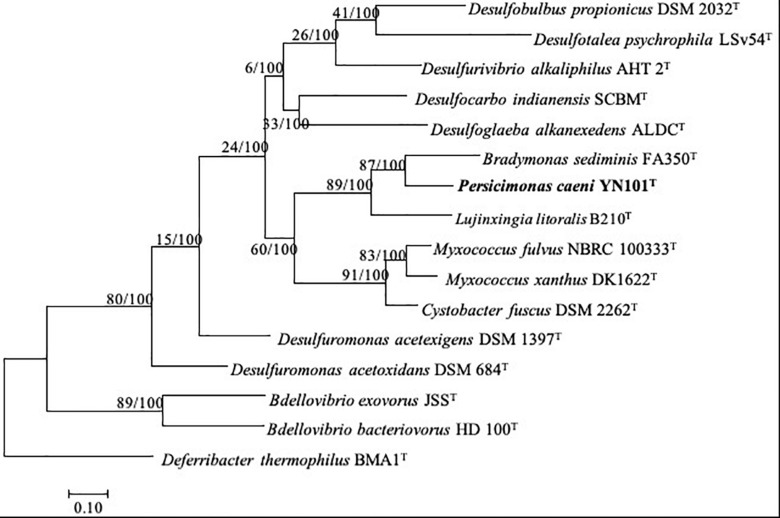
Phylogenomic tree based on up-to-date bacterial core gene sequences of strain YN101^T^ and type strains of related genera within the phylum *Proteobacteria*. The tree was constructed using maximum-likelihood algorithm. Gene support indices (GSIs) and percentage bootstrap values are given at branching points. Bar, 0.10 substitutions per nucleotide position.

The completed genome of strain YN101^T^ was assembled into a single circular chromosome of 8,047,306 bp (144.4-fold coverage) and coded 6009 proteins, similar to the genome size (9,139,763 bp) and gene number (7,181 CDS) of *M. xanthus* DK1622^T^. *B. sediminis* FA350^T^ contained a genome of 5,045,683 bp (Wang et al., 2019), similar in size to the genome of *Bdellovibrio*. Genomes of strain YN101^T^, *B. sediminis* FA350^T^ and *M. xanthus* DK1622^T^ had similar G+C content (mol%) of 60–70%. The dDDH value between strain YN101^T^ and *B. sediminis* FA350^T^ was 13.9%, and the ANI value between the two strains was 74.0%. More details about the genomes of five predators are shown in Table 4. A phylogenomic tree built upon the sequences of 92 core genes (Na et al., 2018) using the maximum likelihood algorithm showed the same taxonomic status of strain YN101^T^ (Figure 3). The taxonomic status of strain YN101^T^ in the phylogenomic tree was consistent with the phylogenetic tree built according to 16S rDNA gene sequence analyses.

**TABLE 4:** Genetic distinction between strain YN101^T^ and each type species of related genera.

Characteristics	1	2	3	4	5
Total length	8,047,306	5,045,683	9,139,763	3,782,950	2,657,893
Gene number	6,072	3,992	7,348	3,597	2,643
Protein coding genes	6,009	3,699	7,181	3,548	2,597
G + C content (mol%)	63.8	61.1	68.9	47.7	41.9
rRNA genes (5S, 16S, 23S)	3, 3, 3	5, 5, 5	4, 4, 4	2, 2, 2	1, 1, 1
tRNA genes	53	54	65	36	33
Key genetic characters					
Biosynthesis of amino acids	D	D	D	D**^§^**	D**^§^**
Biosynthesis of growth factor	D	D	D	D**^§^**	D**^§^**
Type IV pili	C	C	C	C**^§^**	C**^§^**
Flagella	C	D	D	C**^§^**	C**^§^**
Secretion system	T2SS and T3SS	T2SS and T3SS	T4SS and T6SS	T4SS**^§^**	T4SS**^§^**
**dDDH (%)**					
Strain YN101^T^	–				
*B. sediminis* FA350^T^	13.9	–			
*M. xanthus* DK1622^T^	12.9	13.0	–		
*Bd. bacteriovorus* HD100^T^	12.9	12.9	12.9	–	
*Bd. exovorus* JSS^T^	12.9	12.9	12.9	13.1	–
**ANI (%)**					
Strain YN101^T^	–				
*B. sediminis* FA350^T^	74.0	–			
*M. xanthus* DK1622^T^	66.9	66.1	–		
*Bd. bacteriovorus* HD100^T^	64.4	65.0	63.2	–	
*Bd. exovorus* JSS^T^	63.6	63.9	63.1	67.9	–

*1, Strain YN101^T^; 2, *Bradymonas sediminis* FA350^T^; 3, *Myxococcus xanthus* DK1622^T^; 4, *Bdellovibrio bacteriovorus* HD100^T^; 5, *Bdellovibrio exovorus* JSS^T^. D, deficient; C, complete. **^§^** Data from Pérez et al. (2016).*

In addition, comparative genomic analyses between strain YN101^T^ and *B. sediminis* FA350^T^ showed that strain YN101^T^ encoded 189 functional genes more than its neighbor. These genes included hydrogenase metallocentre assembly proteins, ammonium transporter, *β*-ketoadipate enol-lactone hydrolase, dethiobiotin synthetase, chemotaxis regulator (CheY), ATP synthase F_0_ sector subunits, *etc*., as well as many other proteins. Most of these proteins were mainly affiliated with metabolism of many important amino acids, hydrogenase expression/formation, ABC transporters synthesis, benzoate degradation, biotin metabolism, purine metabolism, two-component systems, chemotaxis family and flagellar assembly. Especially these genes encoding synthesis and assembly of flagellum, made strain YN101^T^ behaved more athletic than *B. sediminis* FA350^T^ (Supplementary Figure S2B).

A total of 1,335 common genes in the genomes of five predators could be annotated according to RAST. Strain YN101^T^ and *B. sediminis* FA350^T^ possessed 508 common genes (Supplementary Figure S4), which were affiliated with transportation of oligopeptides, biosynthesis and degradation of several amino acids, and assembly of type IV pili. *M. xanthus* DK1622^T^ and strain YN101^T^ encoded 369 common genes related to transportation of branched-chain amino acids, and adaptation and accommodation to extreme living environments. In addition, 116 common genes contained by all five predators were affiliated with bacterial growth, metabolism and reproduction (Supplementary Figure S4). These genes participated in catabolic pathways of various nutrients, DNA replication, transcription, translation and repair, carbon cycle pathways, nitrogen cycle pathways, and oxidation respiratory chain (Supplementary Figure S5 and Supplementary File S3).

**FIGURE 4:**
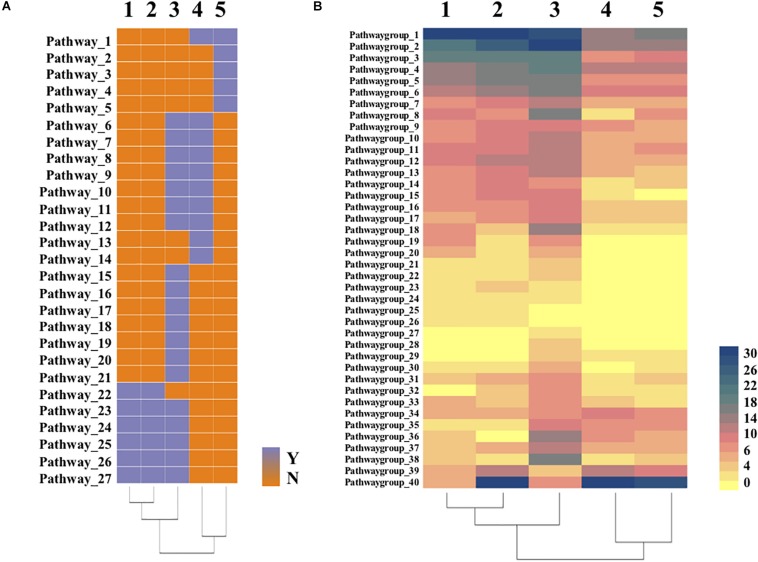
Heatmaps of comparative genomic analysis between strain YN101^T^ and related strains. **(A)** heatmap clustered with 27 pathways according to annotation of the RAST and KEGG; **(B)** heatmap clustered with gene numbers of 40 metabolism groups according to annotation of the RAST and KEGG. 1, Strain YN101^T^; 2, *Bradymonas sediminis* FA350^T^; 3, *Myxococcus xanthus* DK1622^T^; 4, *Bdellovibrio bacteriovorus* HD100^T^; 5, *Bdellovibrio exovorus* JSS^T^.

To further detect differences between *Bradymonadales* and other predators, heatmaps (Figure 4 and Supplementary Files S4, S5) were constructed according to RAST and KEGG annotations. Strain YN101^T^ and *B. sediminis* FA350^T^ lost the complete biosynthesis pathways of threonine, histidine, phenylalanine, valine, leucine, and isoleucine. Biosynthesis metabolism of glycine, serine, methionine and arginine existed in the cells of strain YN101^T^ and *B. sediminis* FA350^T^, however, these amino acids were not *de novo* synthesized by *Bdellovibrio*. *M. xanthus* DK1622^T^ did not synthesize histidine, some aromatic amino acids (tyrosine and phenylalanine), and branched amino acids (valine, leucine and isoleucine). Moreover, the type II and type III secretory system (T2SS and T3SS) of strain YN101^T^ and *B. sediminis* FA350^T^ was more complete than other types of secretory systems, in spite of lacking two transporters named GspS and GspO.

The genetic feature of strain YN101^T^ were summarized as below. Strain YN101^T^ yielded a large genome of 8,047,306 bp, which encoded 189 functional genes more than *B. sediminis* FA350^T^ (according to the analyses of RAST). Its genome encoded many genes affiliated with Type IV pili, Type II secretion system, membrane proteins and transduction proteins, which assisted itself to locate and lyse prey bacteria, and uptake the nutrient from the prey bacteria. The biosynthesis of many important compounds was deficient, which may result in the predation of other bacteria. Genes about flagellum assembly were encoded, thus the strain possessed good athletic ability. Details about genetic features were list in Table 4.

## Discussion

*Bradymonadales* are abundant not only in marine environments, but also in high-salinity inland water environments including saline soil environments (Wang et al., 2015; Bachran et al., 2019; Guo et al., 2019). As a novel member of *Bradymonadales*, strain YN101^T^ has numerous features in common with *B. sediminis* FA350^T^ that differentiate it from other predatory bacterial groups. Cells of strain YN101^T^ are facultatively anaerobic, motile, and prey on a broad spectrum of bacteria. Members of *Bradymonadales* are different facultative predators from *Bdellovibrio* and like organisms (BALOs), and their prey and predatory strategies are distinct from those of myxobacteria. Therefore, comparative analyses about its respiratory pattern, motility and predatory characters with Myxobacteria in *Deltaproteobacteria* and BALOs in *Oligoflexia* were discussed as below.

Many members in the class *Deltaproteobacteria* can utilize sulfate, sulfite, thiosulfate, sulfur or some other inorganic compounds as their terminal electron acceptors (Wang et al., 2015). However, strain YN101^T^ and *B. sediminis* FA350^T^ could utilize nitrate as electron acceptors for anaerobic respiration. Genomic analyses performed *via* RAST indicated that two enzymes, EC 1.7.1.4 and EC 1.7.2.1, were encoded by the genome of strain YN101^T^, which were key to the process of passing electrons from NAD(P)H to nitrate. One flavodoxin reductase (ferredoxin-NADPH reductases) gene, one ferredoxin reductase gene and one arsenate reductase (EC 1.20.4.1) gene were affiliated with anaerobic respiration. Four genes were affiliated with oxidative stress in strain YN101^T^ while two genes were affiliated in the related strain, *B. sediminis* FA350^T^. Strain YN101^T^, rather than *B. sediminis* FA350^T^, possessed the peroxide stress regulator and hydrogen peroxide-inducible gene activator. Therefore, we predict that *Bradymonadales* may possess the evolutionary status of connecting anaerobic and aerobic groups in the class *Deltaproteobacteria*.

Motility is a general property of both obligate and facultative bacterial predators (Lambert et al., 2006; Mendes-Soares and Velicer, 2013). *Bdellovibrio bacteriovorus* runs into the prey during the attack phase propelled by flagellum motility in liquid media, or by slow gliding motility on solid media, and *M. xanthus* groups actively swarm toward the prey using gliding motility (Pérez et al., 2016). As a novel bacterial predator, cells of strain YN101^T^ move by means of flagella and *B. sediminis* FA350^T^ with pili (Supplementary Figure S2A and Table 1), and motility by flagella being more tempestuous than that by pili (Supplementary Figure S2B). Cross-streaking experiments also demonstrated this mode of motility during predation (Supplementary Figure S1A). Cells of strain YN101^T^ and *B. sediminis* FA350^T^ attack prey from the front direction rather than from both sides (Supplementary Figure S2B). Furthermore, based on genetic analyses, the genome of strain YN101^T^ encoded genes affiliated with flagellar motility, which were not encoded by the genome of *B. sediminis* FA350^T^. Among these genes, *flhB*, *fliR*, *flhF*, and *flhA* participated in the biosynthesis of flagella; and *fliN, motA*, and *motB* were responsible for motor switch and rotation of flagella. In addition, gene *flgH* was also found, which could help export the flagellar L-ring proteins across the cell membrane to their destinations in the outer membrane. Apart from the genes described above, genes *fliI*, *flgD*, and *cheY* were also encoded by the genome of strain YN101^T^. Distinctions among the motility of members of *Bradymonadales* give rise to different extents of predation (Supplementary Figure S2B), which may be induced by their genomic differences.

Members of *Bradymonadales* prey on a relatively broad range of bacterial types. Thus, we selected 4 representative species (Supplementary Figure S1) for further analyses. *Woeseiaceae*, abundant in both coastal and deep-sea sediments (Bacosa et al., 2018), oxidize sulfite and degrade hydrocarbons, suggesting that members of *Woeseiaceae* are functional in carbon, sulfur and nitrogen cycling in diverse marine sediment types (Mußmann et al., 2017; Bacosa et al., 2018). Especially, some members of the *Woeseiaceae* also have the genetic potential for chemolithoautotrophy powered by sulfur or hydrogen oxidation (Mußmann et al., 2017). Meanwhile, *Algoriphagus* is widely distributed in the marine environment. Studies report certain members of *Algoriphagus* can encode a carotenoid synthesis gene cluster (Tao et al., 2006), indicating *Algoriphagus* bacteria may have influences on marine animal cell biology (Alegado et al., 2012). *Nocardioides* is one important representative taxon of actinomycetes, which can produce antibiotics (Matson and Bush, 1989; Ortiz-López et al., 2018), and its members inhabit different natural surroundings, including soil, cave, seawater and desert (Zhang et al., 2016; Khan et al., 2017; Lu et al., 2017; Wang et al., 2017). These three species represented bacterial groups associated with sulfur metabolism, pigments and antibiotic production. Moreover, predator-prey interactions play important roles in the cycling of marine organic matter. For instance, some bacterial predators bind to the glycan strands of Gram-stain positive bacterial peptidoglycan (PG) and degrades the PG peptide chains, leading to cell death (Tang et al., 2020). The released nutrients, including PG-derived D-amino acids, can then be utilized by these predators for growth. Additionally, for the formation of biofilm during predation, the order *Bradymonadales* may play important roles in structuring the marine particle- and surface-associated microbial community composition (Dang and Lovell, 2016). On surfaces and particles, higher encounter rate may accelerate predation rate other than these individual microbes free-living in seawater. Therefore, predation on these three diverse groups of bacteria indicated that the order *Bradymonadales* may play a significant role in regulating marine microbial communities and the global biochemical cycle.

Furthermore, we found that both strain YN101^T^ and *B. sediminis* FA350^T^ showed different predatory behaviors to different species of the same genus. For instance, two predators could prey on *A. marinus* am2^T^ and *N. gilvus* XZ17^T^ but not on *A*. *resistens* NH1^T^ and *N. albus* JCM 3185^T^. Cross-streaking experiments qualitatively demonstrated the predation behavior of two predators but could not quantitatively measure how the biomasses of predators and prey change during this process. Therefore, *A. marinus* am2^T^ was chosen as prey for strain YN101^T^ and *B. sediminis* FA350^T^ for absolute quantification by fluorescent quantitative PCR. Three time periods were representative of prophase, metaphase and anaphase of the predation process, which were demonstrated by colony-counting results using the plate culture technique (Supplementary Figure S6). As shown in Figure 2, two co-cultured predators killed *A. marinus* am2^T^ and their biomasses increased. Furthermore, their biomass increases were greater than those observed in the mono-cultured control groups. Cell numbers of mono-cultured prey indicated *A. marinus* am2^T^ grew normally without predatory stress. Therefore, we considered that the decrease of *A. marinus* am2^T^ and increases of strain YN101^T^ and *B. sediminis* FA350^T^ in the co-cultured system were caused by the latter two microorganisms preying on *A. marinus* am2^T^. Deviations in the extraction of the sample genome might have given rise to a small reduction of co-cultured strain YN101^T^ at 68 h.

As shown in Figure 4A, all five predators lost their ability to *de novo* synthesize several amino acids. Some obligate epibiotic predators depend on prey strictly because their genomes do not encode genes to synthesize certain amino acids or to import them directly from the environment (Wang et al., 2011; Soo et al., 2015). Predatome results exhibited deficiencies in riboflavin and amino acids biosynthesis, suggesting that predators obtain these molecules from their prey (Pasternak et al., 2013). In other words, predation is one important approach by these organisms to uptake certain kinds of amino acids. Figure 4B demonstrated the differences in gene numbers of 40 metabolism groups from the five predators. Strain YN101^T^ and *B. sediminis* FA350^T^ had more complete pathways than *Bdellovibrio* for the biosynthesis of some amino acids, fatty acids and carbohydrates. However, biosynthesis of threonine, valine, leucine, isoleucine, lysine, histidine, phenylalanine, and tyrosine were still incomplete, which may induce to the predation of *Bradymonadales*.

Strain YN101^T^ and *B. sediminis* FA350^T^ encoded many genes affiliated with secretion system (including T2SS and T3SS) and signal transduction system. Moreover, type IV pilus (T4P) were also found in the genomes of *Bradymonadales*. *Bradymonadales* might invade Gram-negative bacteria during the predation process, and it was necessary to locate the prey by sliding T4P on the surface of the substrate. T4P also consisted the attachment structure to prey cells. Secretion system might participate in the process of predation by secreting antibiotics and hydrolases to kill or lyse the prey cells (Pérez et al., 2016). T3SSs might form channels that cross the bacterial envelope and the prey cell membrane, which enable bacteria to inject numerous effector proteins into the prey cell cytoplasm and establish trans-kingdom interactions with diverse prey (Deng et al., 2017). T2SS could hypothetically be recombined with proteins replacing GspS and GspO, assisting pathogens to infect hosts. GspS is one compound of the EnvZ/OmpR two-component system that mediates the osmotic stress response in a number of Gram-stain negative bacteria, while GspO is a leader peptidase (Gueguen et al., 2013; Zhang et al., 2013). Therefore, we predicted strain YN101^T^ and *B. sediminis* FA350^T^ were probably able to prey on bacteria with participation from secretion system.

In conclusion, chemotaxonomic, physiological, and biochemical analyses distinguished strain YN101^T^ from its related strain, *B. sediminis* FA350^T^, and strain YN101^T^ is a novel predator in *Deltaproteobacteria*. Studies on this novel predatory group have great potential value for exploring microbial behavior and ecology. Our work lays the foundation for further study of *Bradymonadales* in the future.

### Description of *Persicimonas* gen. nov.

*Persicimonas* (*Per’si.ci.mo’nas.* Gr. adj. *persicus* of peach; Gr. n. *monas* a unit, monad; N.L. fem. n. *Persimonas*, a peach-colored monad).

Cells are Gram-stain negative, rod-shape, facultatively anaerobic and motile. Oxidase negative and catalase positive. The major polar lipid profile consists of diphosphatidylglycerol (DPG), phosphatidylglycerol (PG), phosphatidylethanolamine (PE) and unidentified aminophospholipid (APL). The predominant cellular fatty acid is iso-C_17:0_. The sole ubiquinone is MK-7. The type species is *Persicimonas caeni*.

### Description of *Persicimonas caeni* sp. nov.

*Persicimonas caeni* (cae’ni. L. gen. n. *caeni* of sludge) Cells are 0.3–1.0 μm in width and 1.5–5.0 μm in length. Colonies were circular or irregular, pink-colored, about 1 mm diameter, and with entire and transparent edges. Growth occurs at 15–50°C (optimum 37°C) and pH 6.5–9.0 (optimum 8.0–8.5) with 1–10 % (w/v) NaCl (optimum 3–4 %). Nitrate cannot be reduced to nitrite. Positive for alkaline phosphatase, esterase lipase (C8), leucine arylamidase, acid phosphatase and naphthol-AS-BI-phosphohydrolase, but negative for esterase (C4), lipase (C14), valine arylamidase, cystine arylamidase, trypsin, *α*-chymotrypsin, *α*- and *β*-galactosidase, *β*-glucuronidase, *α*- and *β*-glucosidase, *N*-acetyl-*β*-glucosaminidase, *α*-mannosidase and *α*-fucosidase. Arginine hydrolysis, gelatinase and D-glucose oxidation were positive. Negative for *β*-galactosidase, lysine decarboxylase, omithine decarboxylase, citrate utilization, H_2_S production, urease, tryptophan deaminase, indole production, acetoin production, D-mannitol oxidation, inositol oxidation, D-sorbitol oxidation, L-rhamnose oxidation, D-saccharose oxidation, D-melibiose oxidation, amygdaline oxidation, and L-arabinose oxidation tests. Acids are produced with the substrates D-ribose, D-fructose, L-sorbose, esculin, D-xylose, D-tagatose and potassium 5-ketogluconate. D-Glucuronic, glucuronamide, *α*-keto-glutaric and acetoacetic are oxidized.

The type strain, YN101^T^ (=KCTC 72083^T^ = MCCC 1H00374^T^) was isolated from a marine solar saltern (122°14′E, 36°54′N) on the coast of Weihai, PR China. The DNA G + C content of the type strain is 63.8 mol %. The GenBank accession number for the 16S rRNA gene sequence of *Persicimonas caeni* YN101^T^ is MH718301. The complete genome sequence of strain YN101^T^ has been deposited at GenBank under the accession CP041186.

## Data Availability Statement

The GenBank accession numbers for the 16S rRNA gene sequence and the whole-genome project of strain YN101^T^ are MH718301 and CP041186, respectively.

## Author Contributions

SW, DM, and Z-JD designed the experiments. SW carried out the experiments and analyzed the experimental results. SW wrote the manuscript.

## Conflict of Interest

The authors declare that the research was conducted in the absence of any commercial or financial relationships that could be construed as a potential conflict of interest.

## Footnotes

1 https://blast.ncbi.nlm.nih.gov/Blast.cgi

2 http://www.ezbiocloud.net

3 sg.idtdna.com/PrimerQuest

4 www.originlab.com

5 https://github.com/aquaskyline/SOAPdenovo2

6 www.ezbiocloud.net/tools/ubcg

7 ggdc.dsmz.de

8 rast.nmpdr.org

9 www.genome.jp/kegg/

10 hemi.biocuckoo.org

11 www.ncbi.nlm.nih.gov

## Abbreviations

dDDH: digital DNA–DNA hybridization
GGDC: genome-to-genome distance
HPLC: High Performance Liquid Chromatography
KCTC: Korean Collection for Type Cultures
KEGG: Kyoto Encyclopedia of Genes and Genomes
MA: Marine agar 2216
MB: Marine broth 2216
MCCC: Marine Culture Collection of China
MEGA: Molecular Evolutionary Genetics Analysis
MIDI: Microbial Identification System
PG: peptidoglycan
RAST: Rapid Annotation using Subsystem Technology
TLC: Thin layer chromatography
UBCG: Up-to-date bacterial core gene.
