# Supplementary material for: Persicimonas caeni gen. nov., sp. nov., the Representative of a Novel Wide-Ranging Predatory Taxon in Bradymonadales
Source: Front Microbiol. 2020 Apr 22;11:698. doi: 10.3389/fmicb.2020.00698 (PMC7188933; doi:10.3389/fmicb.2020.00698)
Supplement: Supplementary file 1 [file Presentation_1.pdf]

Supplementary Material

Persicimonas caeni gen. nov., sp. nov., the representative of a novel wide-ranging predatory taxon in _Bradymonadales_

Shuo Wang,[1] Da-Shuai Mu,[1,2] and Zong-Jun Du[1,2]*

[1]Marine College, Shandong University, Weihai, Shandong, 264209, China

[2]State key Laboratory of Microbial Technology, Shandong University, Qingdao, Shandong, 266237, China

Supplementary File S1. Gene information and condition of fluorescent quantitative realtime-PCR

Supplementary File S2. Original data of fluorescent quantitative realtime-PCR

Supplementary File S3. Roles of 116 common genes contained by all five predators

Supplementary File S4. Genomic differences between *Bradymonadales* and other predators according to annotation of RAST

Supplementary File S5. Genomic differences between *Bradymonadales* and other predators according to annotation of KEGG

Supplementary Fig. S1

Supplementary Fig. S2

Supplementary Fig. S3

Supplementary Fig. S4

Supplementary Fig. S5

Supplementary Fig. S6

Supplementary Fig. S7

Supplementary Table S1

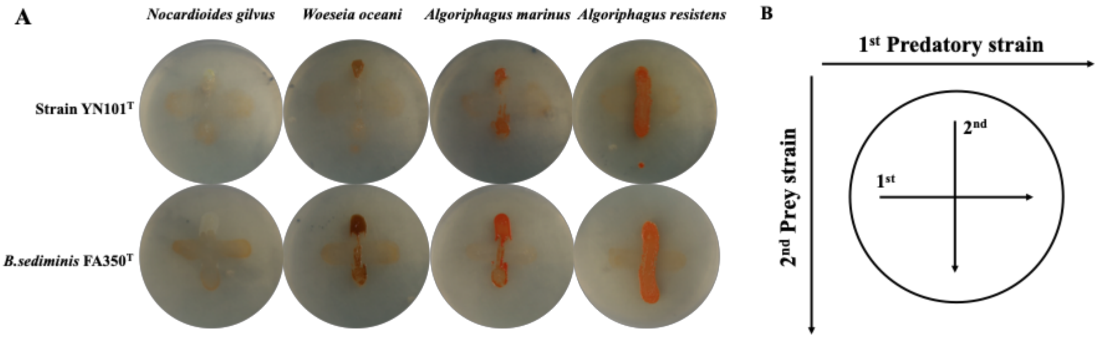

Supplementary Fig. S1 Cross-steaking incubation tests of *Persicimonas caeni* YN101^T and *Bradymonas sediminis* FA350^T with different test strains. The plate was incubated until the growth of both predator and prey could be indicated by visible lawn. Cells of predators were carried and co-inoculated with the test bacteria on the second line when the test bacteria inoculation passed through the first line. Once the test bacteria were prey, cells of predators killed them and grew in the second line mixed with residual and survival cells of test bacteria.

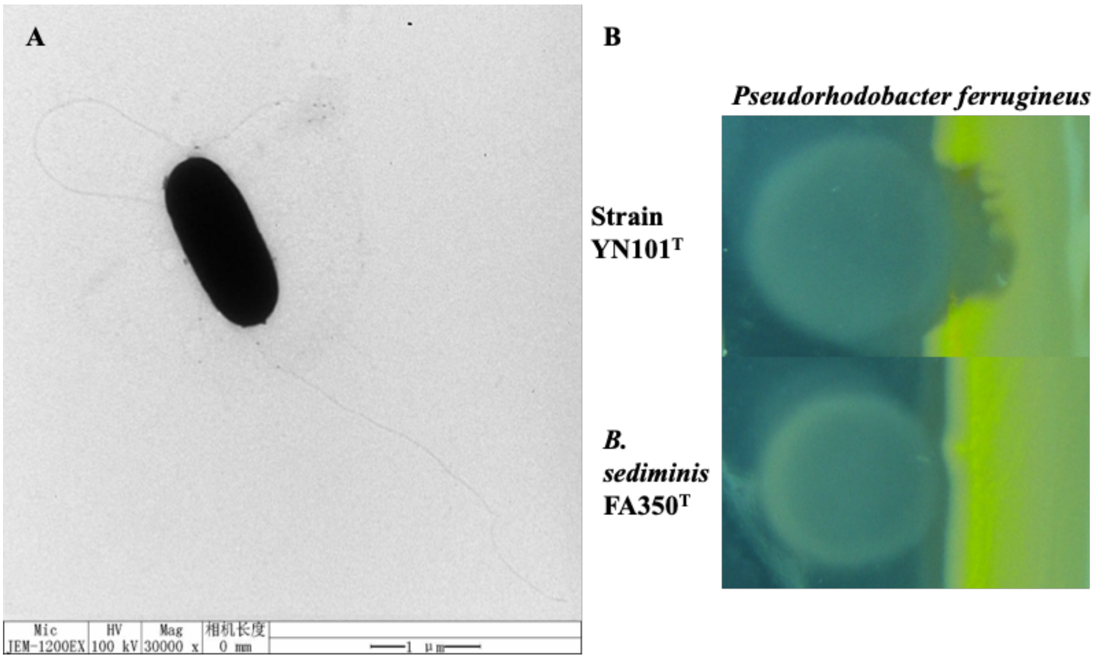

Supplementary Fig. S2 (A) Transmission electron micrographs of *Persicimonas caeni* YN101^T; Bars, 1 μm. (B) Different motility means of strain YN101^T and *B. sediminis* FA350^T when predating; Nikon SMZ 745T (100 ×).

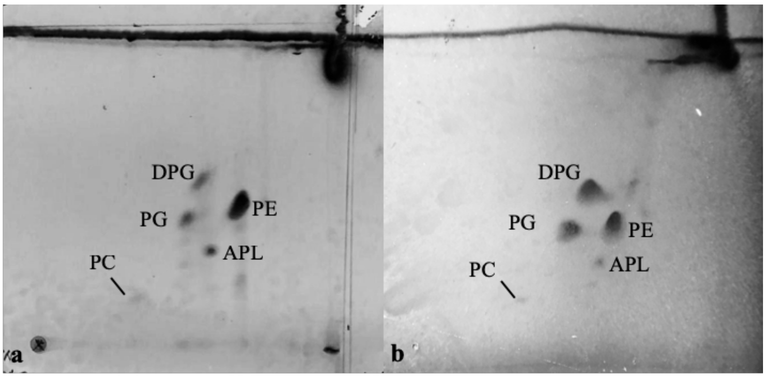

Supplementary Fig. S3 Two-dimensional TLC plate of lipids extracted from *Persicimonas caeni* YN101^{T} and related strain. a, *Persicimonas caeni* YN101^{T}; b, *Bradymonas sediminis* FA350^{T}. The plate was sprayed with 5% (v/v) molybdatophosphoric acid to show all lipids present. PG, phosphatidylglycerol; PE, phosphatidylethanolamine; DPG, diphosphatidylglycerol; APL, unidentified aminophospholipids; PC, unidentified phosphatidylcholine.

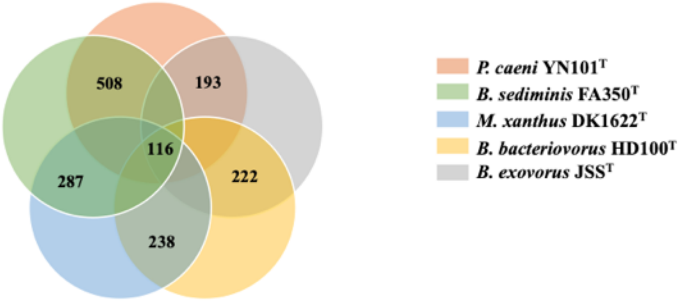

Supplementary Fig. S4 Venn diagram of comparative genomic analysis between strain YN101^{T} and type species in related genera according to annotation of the RAST.

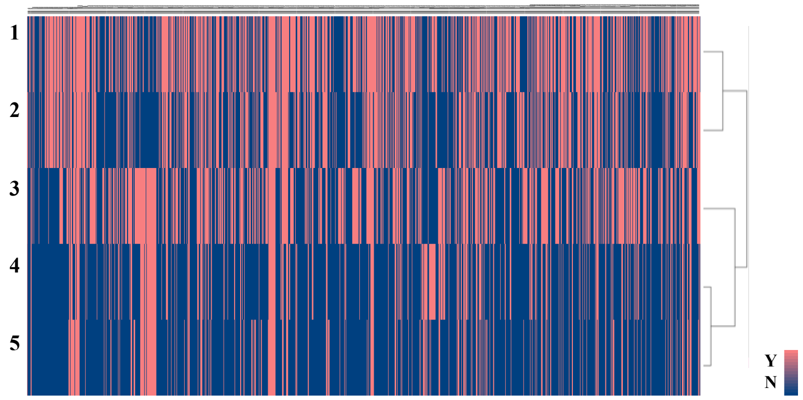

Supplementary Fig. S5 Heatmaps of comparative genomic analysis between strain YN101^T and type species in related genera according to annotation of the RAST. Descriptions of functional genes were available in Supplementary File S1.

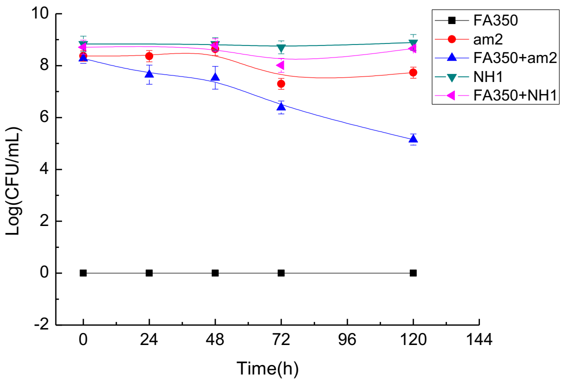

Supplementary Fig. S6 Biomass of *B. sediminis* FA350^T and prey *Algoriphagus marinus* am2^T, as well as non-prey *Algoriphagus resistens* NH1^T quantified with the method of colony-counting by plate culture technique. Each design was repeated three times, and the result was the average of the three replicates.

1

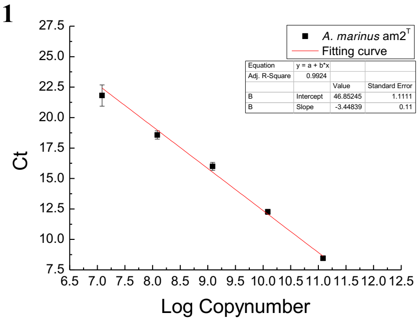

2

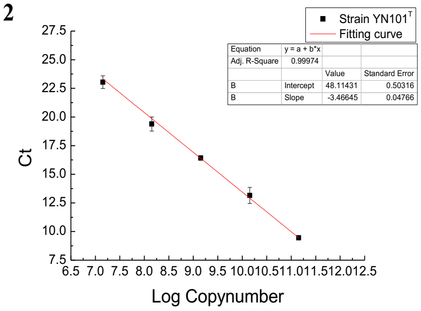

3

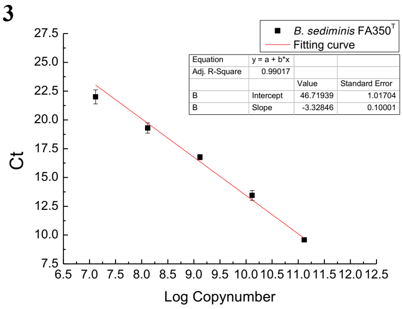

Supplementary Fig. S7 The quantitative standard curves of three specific genes for absolute quantification by RT-PCR. 1, *Algoriphagus marinus* am2^{T}; 2, *Persicimonas caeni* YN101^{T}; 3, *Bradymonas sediminis* FA350^{T}. Each design was repeated three times, and the result was the average of the three replicates.

Supplementary Table S1 Fatty acid contents that distinguish *Persicimonas caeni* YN101^T from related species.

1, *Persicimonas caeni* YN101^T; 2, *Bradymonas sediminis* FA350^T.

All data are from this study. Fatty acids amounting to <1.0 % in both strains studied are not shown.

tr, Trace, < 1.0 %; ND, not detected.

**Fatty acid**	**1**	**2**
**Straight-chain fatty acids**		
$C_{16:0}$	1.1	ND
$C_{17:0}$	9.1	tr
**Branched fatty acids**		
iso-$C_{15:0}$	9.8	**40.9**
iso-$C_{16:0}$	4.8	tr
iso-$C_{17:0}$	**54.8**	**30.7**
iso-$C_{15:1}$ $\omega 9c$	1.8	2.1
iso-$C_{17:1}$ $\omega 10c$	ND	**13.7**
**Unsaturated fatty acids**		
$C_{15:1}$ $\omega 8c$	2.1	ND
$C_{16:1}$ $\omega 5c$	2.8	ND
$C_{16:1}$ $\omega 9c$	0.8	1.8
$C_{17:1}$ $\omega 6c$	1.3	ND
$C_{17:1}$ $\omega 7c$	1.2	ND
$C_{17:1}$ $\omega 9c$	2.3	ND
**Summed Feature 4***	1.6	ND
**Summed Feature 5***	tr	2.6

*Summed features represent groups of two or three fatty acids that cannot be separated by the Microbial Identification System. Summed feature 4 comprises iso-$C_{17:1}$ I and/or anteiso-$C_{17:1}$. Summed feature 5 comprises ante-$C_{18:0}$ and/or $C_{18:2}$ $\omega 6,9c$.
